# Severe Fetal Distress and Umbilical Cord Strangulation

**DOI:** 10.1155/2011/645487

**Published:** 2011-07-18

**Authors:** Giovanni Larciprete, Carlotta Montagnoli, Paolo Fusco

**Affiliations:** ^1^Department of Obstetrics and Gynecology, Fatebenefratelli Isola Tiberina Hospital, Rome, Italy; ^2^AFaR, Fatebenefratelli Association for Research, Fatebenefratelli Isola Tiberina Hospital, Rome, Italy; ^3^School of Specialization in Obstetrics and Gynaecology, Tor Vergata University, Rome, Italy

## Abstract

We describe an extreme case of amniotic band syndrome, presented with fetal stress during labor and associated with strangulation of umbilical cord.

## 1. Introduction

The amniotic band sequence is a rare syndrome which includes several congenital deformities, (usually at limbs or digits) caused by entrapment of these parts in fibrous amniotic bands [1]. The clinical manifestations vary from extremity amputations to anencephaly or fetal death secondary to strangulation of umbilical cord [2]. 

We report an extreme case of fetal distress associated with entrapment and strangulation of umbilical cord within an amniotic band, resulting in live born thanks to the careful evaluation of the parameters of fetal health in labor.

## 2. Case Presentation

On November the 9th, 2010, a 39-year-old women, gravida 2 para 1, at 41-week gestation, was admitted to our Department of Obstetric with decreased variability in fetal heart rate monitoring associated with oligohydramnios. The ultrasound scan performed at admission showed a vertex presenting fetus with an estimated weigth of 2800 g, reduced amniotic fluid index (AFI 40), and normal Doppler PI of umbilical artery. Her previous obstetrical and medical history was unremarkable, and her current pregnancy was ordinary. 

**Figure 1:**
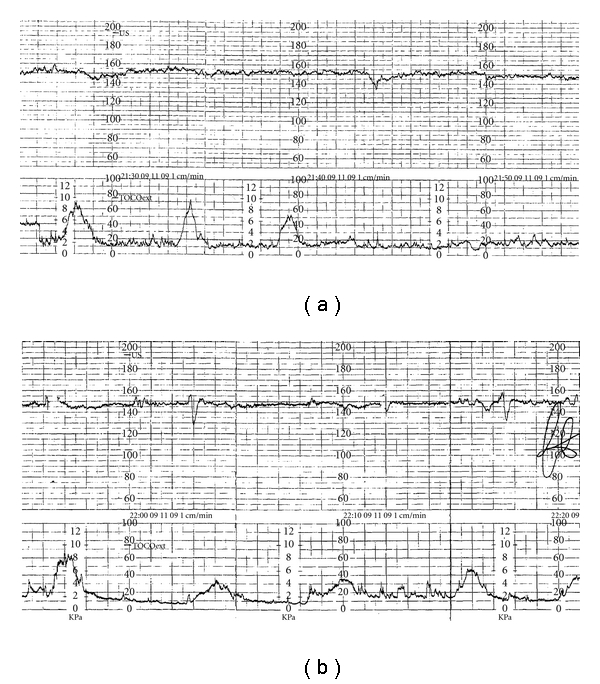
Fetal heart rate monitoring: (a,b). Persistent decreased variability (<5 bpm).

**Figure 2:**
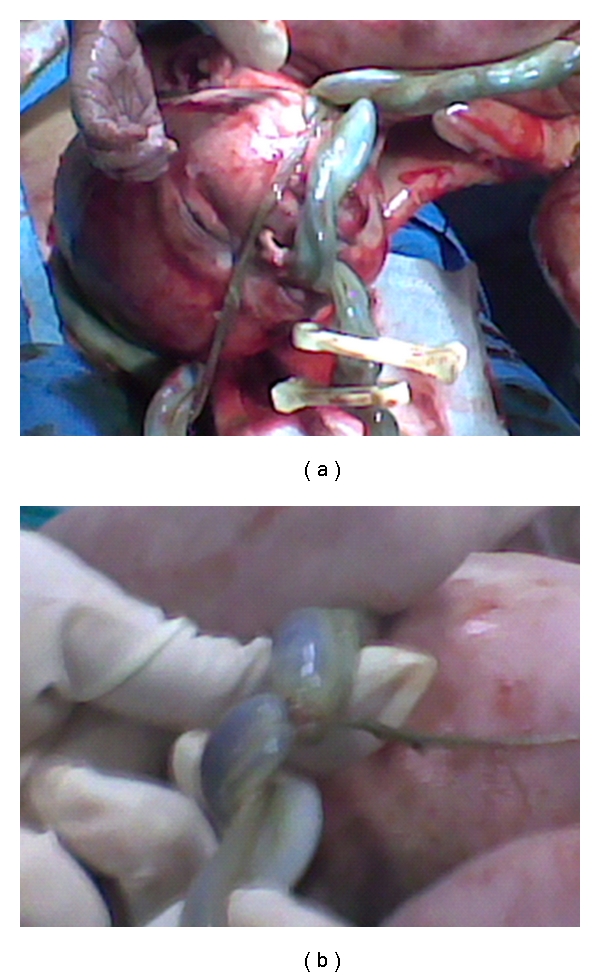
Umbilical cord strangulation: (a) Entrapment of umbilical cord by amniotic band. (b) Visualization of amniotic band.

Within 40 minutes of admission, an induction of labor with Oxytocin 5 UI was performed under cardiotocography monitoring. Two hours after the induction, we still observed a reduced variability in fetal heart rate from cardiotocography (amplitude range of 5 beats/minute) with sporadic late decelerations (Figures 1(a) and 1(b)), then we proceeded to amniorrhexis which revealed meconium-stained amniotic fluid. Therefore, a cesarean section was performed for acute fetal distress, since spontaneous vaginal delivery was not imminent. An asphyxiated, 2620 g female newborn was delivered, with Apgar score 2 and 8 at 1 and 5 minutes, respectively. The newborn had cardiac activity, but she breathed after ventilation. Unfortunately, we do not have any data about fetal or neonatal blood pH or BE, because in that circumstance, the blood sample clotted early before allowing the measure. The examination of the placenta and the umbilical cord revealed an amniotic band causing entrapment and strangulation of part of the umbilical cord (Figures 2(a) and 2(b)).

The newborn did not show other disorders due to amniotic band sequence. Both mother and neonate were discharged from hospital after 3 days without complications. The neonate was followed up and remained in good health after 1 year of delivery.

## 3. Discussion

The amniotic band sequence occurs in approximately 1/2000–1/15000 live births [2], but the presence of amniotic band is associated to 1%-2% of fetal malformations [3], and 10% of this congenital syndrome include umbilical cord strangulation [4]. 

Although the mechanism underlying the syndrome is unknown, however, the accepted hypothesis is that an early rupture of the amniotic sac leads to the formation of amniochorionic mesodermal bands [5]. The amniotic band determines clinical manifestations through entanglement by amniotic band, interference with normal development, and disruption secondary to cleavage of structure already developed normally [6]. However, these mechanisms are not able to explain all types of malformations.

Also, the type of deformities depends on time of amniotic rupture. It is assumed that the minor defect of extremities occur in late period, while an early amniotic rupture leads to the most severe visceral disruption, determining the different prognosis.

The prenatal diagnosis by ultrasound of the amniotic band is often difficult, and frequently the simultaneous presence of different congenital deformities suggests the presence of an amniotic band syndrome. 

Unfortunately, in our case, the diagnosis of amniotic band was not determined during pregnancy. Only rare cases of strangulation of umbilical cord by amniotic band have been described in the literature, most of whom were stillborn [4, 7]. The cause of fetal death during labor is that the contraction intense enough to stop the blood flow through the umbilical cord constriction by amniotic band, determining severe fetal hypoxia [8].

Instead, we report a case of constricted umbilical cord by amniotic band, but fortunately, in this case, we intervened in time and despite the severe distress, the fetus was alive and actually is in good health.

Despite the fact that during labor the different unexpected umbilical cord lesions can occur, as we have already described in other reports [9, 10], this case suggests that the ultrasound diagnosis of amniotic band allows an attempted delivery and can explain signs of severe fetal distress at an early stage, leading the obstetricians to carefully evaluate the best route for a safe delivery.
